# Tuberculosis‐Associated Paradoxical Immune Reconstitution Inflammatory Syndrome in an HIV‐Negative Patient Presenting With a Recurrent Pleural Effusion Managed With Prednisolone: A Case Report

**DOI:** 10.1002/ccr3.71843

**Published:** 2026-01-11

**Authors:** Selam Bogale Gissa, Alemayehu Girma Gemechu, Selamawit Tilahun

**Affiliations:** ^1^ Infectious Disease Unit, Internal Medicine Department, College of Health Sciences Addis Ababa University Addis Ababa Ethiopia; ^2^ Department of Internal Medicine Yekatit 12 Hospital Medical College Addis Ababa Ethiopia; ^3^ Pulmonary and Critical Care Unit, Internal Medicine Department St. Paul Hospital Millennium Medical College Addis Ababa Ethiopia

**Keywords:** case report, Ethiopia, HIV negative, IRIS, treatment, tuberculosis

## Abstract

Non‐HIV‐infected people with pulmonary tuberculosis can develop paradoxical worsening of lung conditions, including massive pleural effusion, which at times requires multiple drainage. This paradoxical immune reconstitution inflammatory syndrome can successfully be treated with a short course of steroids, resulting in symptom, quality of life, and imaging improvements.

## Graphical abstract

Chest CT scan 1 month after starting TB treatment demonstarting left side massive pleural effusion.

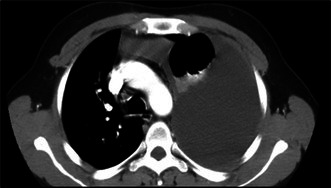

## 1 Introduction

Initiation of drugs for the treatment of tuberculosis (TB) results in marked clinical improvement; however, in some patients, a paradoxical symptomatic and/or radiologic worsening occurs. This is known as immune reconstitution inflammatory syndrome (IRIS). IRIS occurs due to a heightened immune response and is well recognized and described in human immunodeficiency virus (HIV) infected individuals initiated on combined antiretroviral drugs. IRIS in the setting of HIV could be either paradoxical, which is the worsening of a known opportunistic infection like TB, or unmasking, that is, the appearance of a previously unrecognized infection. Paradoxical worsening after initiation of anti‐TB drugs in non‐HIV‐infected individuals is known to occur but, unlike in HIV‐infected patients, a formal consensus definition and treatment approach is lacking [1].

The prevalence of paradoxical TB IRIS in non‐HIV‐infected individuals ranges from 1.5% to 23% [2, 3]. We report TB IRIS in an HIV‐negative patient presenting with worsening pleural effusion despite recurrent therapeutic drainage and successfully treated with a short course of steroid.

### 1.1 Case History and Examination

A 37‐year‐old male presented with progressive worsening of shortness of breath with left‐sided pleuritic chest pain. He also has stabbing upper back pain. He has gradually increasing fatigue and palpitation restricting his usual activity. He has no body swelling. He does not smoke or drink alcohol. He was vaccinated for COVID‐19 and had taken BCG and hepatitis B vaccines. He has no household TB contact; however, he is a physician who works at medical outpatients in a TB endemic setting, making him vulnerable to exposure. Four weeks prior to the current presentation, he was diagnosed with TB after presenting with chest pain exacerbated by inhalation and cough with a small amount of whitish sputum, with associated dyspnea, night sweats, and weight loss. Treatment was commenced July 25, 2022 with standard four‐drug TB treatment with rifampicin, isoniazid, ethambutol, and pyrazinamide; despite adherence, there was no clinical improvement. He had no treatment for TB before the current one. He was in respiratory distress at presentation but afebrile, and oxygen saturation was above 90%, and there was decreased breath sound and dullness on the left posterior half of the chest. Other systems were unremarkable.

In the emergency unit, he had pleural fluid drained, resulting in symptomatic improvement. He returned after 2 days with a similar complaint and had to have an additional two therapeutic pleural taps of 1 L 3 days apart from the left side.

### 1.2 Differential Diagnosis, Investigations, and Treatment

Key differential diagnoses for this case include MDR‐TB, para‐pneumonic effusion, malignant pleural effusion, TB pleurisy, and TB‐IRIS. TB‐IRIS is the likely cause in this case mainly because the other differentials are less likely; initial sputum examination showed TB, which was rifampicin sensitive. He is young with no history of smoking and other site complaints or physical findings, making primary and metastatic malignancies of the pleura less likely. A protracted course with a massive effusion is also less common in pneumonia with para‐pneumonic effusion.

**FIGURE 1:**
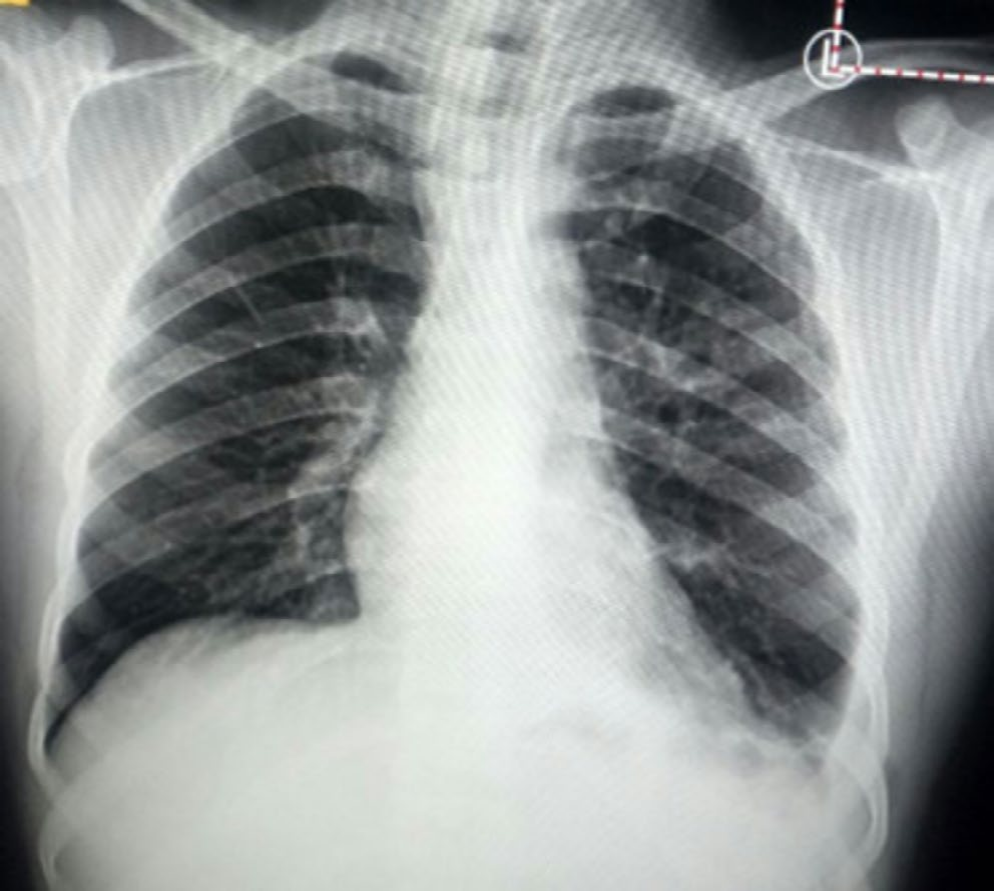
Chest X ray at TB diagnosis 20 July 2022 showing left costophrenic angle obliteration.

Initial tests at diagnosis of TB showed an erythrocyte sedimentation rate (ESR) of 10 mm/h (reference < 20 mm/h). The complete blood count (CBC), renal function test, and liver enzymes were within the reference range. HIV test was negative. Posterio‐anterior chest x‐ray at TB diagnosis on July 20, 2022 (Figure 1) revealed blunting of the left costophrenic angle; chest ultrasound confirmed the presence of minimal left‐sided pleural effusion. Gene Xpert from sputum was positive for TB, which was rifampicin sensitive. During the current presentation four weeks after initiation of anti‐TB pleural fluid was sent for analysis and revealed a proteinaceous background with dominant lymphocytes, few mesothelial cells, and macrophages with no malignant cells. Pleural fluid for AFB staining, Gene Xpert, TB culture were negative, making TB pleurisy less likely but not unlikely as these tests are not 100% sensitive. Gram stain and bacterial culture of the pleural fluid have not revealed any organism. ADA test was not done as it was not available in the hospital at the time and is available only in a few private laboratories in the country.

**FIGURE 2:**
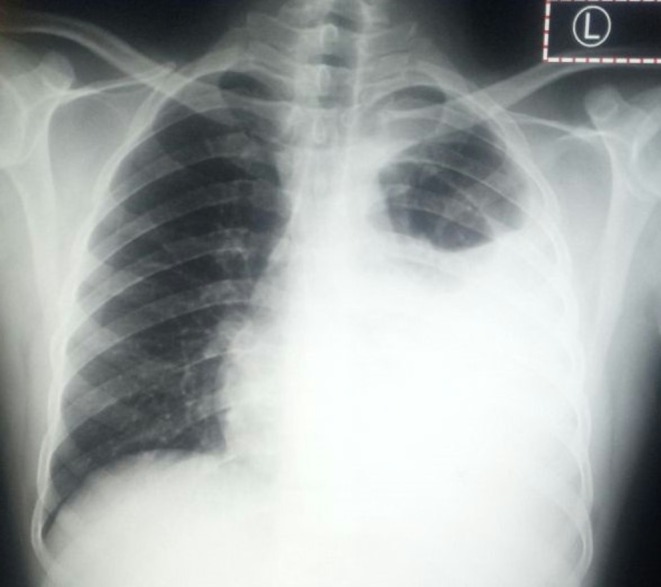
Chest X ray 1 month after starting TB treatment 19 August 2022 with evidence of marked increase in left pleural effusion.

**FIGURE 3:**
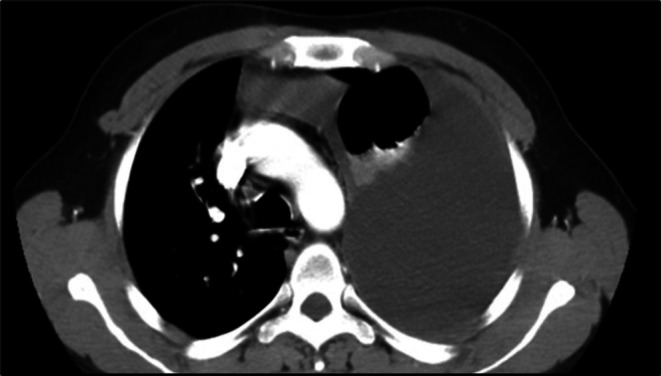
Chest CT scan 1 month after starting TB treatment 26 August 2022 demonstrating the left side massive pleural effusion.

As the fluid kept on recollecting despite repeated therapeutic drainages and the repeated chest x‐ray (August 19, 2022), a month after the initiation of TB treatment showed a marked increase in the level of pleural fluid on the left side (Figure 2) chest CT scan was done on August 26, 2022, and showed left side massive pleural effusion with features of empyema, left upper lobe perihilar area decreased enhancement with mildly enlarged hilar and mediastinal lymph nodes (Figure 3). After the CT scan, pleural biopsy was planned but the client did not want it due to fear of complications. Additional tests were done, including tumor markers and abdominal ultrasound, which were normal. Repeat pleural fluid cytology was like the initial. Pleura fluid cell count was 2450 cells/μL with 85% lymphocytes. C‐reactive protein (CRP) was 15.2 mg/L (reference < 10 mg/L). Echocardiography was normal and magnetic resonance image (MRI) of the thoracic vertebrae was unremarkable except for the massive pleural effusion.

**FIGURE 4:**
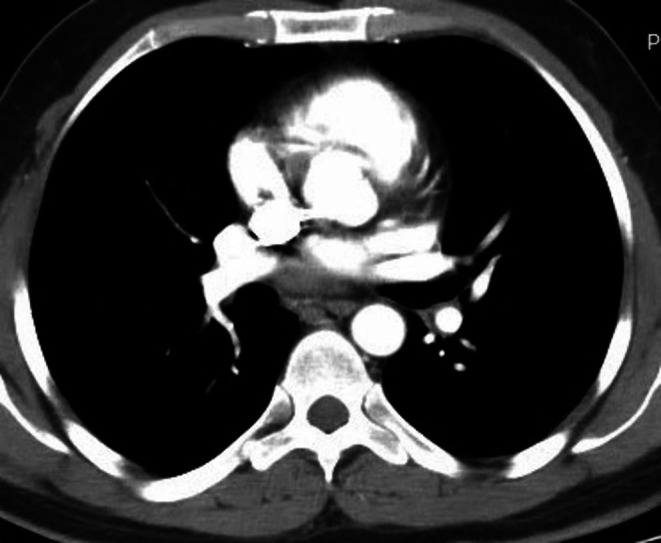
Chest CT after completion of TB treatment 21 February 2023 showing interval disappearance of the left side pleural effusion.

As other differential diagnoses, including malignant effusion were deemed less likely (clinically, imaging and laboratory tests, including two cytology examinations of the pleural fluid) TB IRIS was considered the most likely cause of the repeated massive pleural effusion, hence prednisolone 40 mg PO daily was started. The prednisolone was tapered by half every 2 weeks and discontinued; this was done by recommendation of a pulmonologist with experience in TB management, as there is no specific guideline or text book recommendation. He was also initiated on cotrimoxazole for pneumocystis pneumonia (PCP) prophylaxis. Three more therapeutic pleural drainage of about 1 L per session were done. Subsequently, the shortness of breath decreased. Repeat chest CT scan 12 days later (on September 8, 2022) showed: left upper lobe focal mass like consolidation with cavitary changes and adjacent fibrotic bands; adjacent nodules; left side moderate pleural effusion; mildly enlarged left hilar and medastinal lymph nodes. The TB treatment was completed as per the national guidline, and prednisolone was tapered and discontinued after a total of 2 months. After a total of 6 months the TB treatment was discontinued and follow‐up chest CT on February 21, 2023, revealed left upper lobe posterior segment focal fibrosis and traction bronchiectasis with volume loss‐sequele of TB, the rest of the lungs bilaterally have normal parenchymal attenuation, no mass, no nodule no pleural effusion, and no lymphadenopathy (Figure 4). Two months after finishing TB treatment, he had no symptoms and resumed work and usual level of physical activity.

### 1.3 Outcome and Follow‐Up

Following treatment with steroid, he had marked symptomatic improvement. After a total of 6 months since initiation the TB treatment was completed and follow‐up chest CT revealed left upper lobe posterior segment focal fibrosis and traction bronchiectasis with volume loss‐sequele of TB, the rest of the lungs bilaterally have normal parenchymal attenuation, no mass, no nodule, no pleural effusion, and no lymphadenopathy (Figure 4). Two months after finishing TB treatment, he had no symptoms and resumed work and his usual level of physical activity. It has been 2 years since he initially presented and he has no symptoms currently and is in a good state of health.

## 2 Discussion

Host factors, especially the immune system, and bacterial factors like the load of the bacilli, with the presence of certain risk factors like younger age, male gender, and use of biologic agents, are proposed in the pathogenesis of TB IRIS. However, the detailed mechanism and definition of TB IRIS in HIV‐uninfected individuals is not fully defined. Though there seems to be a paucity of data, the following four factors should be fulfilled to diagnose TB IRIS:

1. Initial improvement of TB‐related symptoms and/or radiographic findings after adequate anti‐TB treatment for a certain time;
2. Paradoxical deterioration of TB‐related symptoms and/or radiologic findings at the primary or at new locations during or after anti‐TB treatment;
3. Absence of conditions that reduce the efficacy of anti‐TB drugs (e.g., poor compliance, drug malabsorption, drug side effects);
4. Exclusion of other possible causes of clinical deterioration.

Protein clinical presentation of TB IRIS have been reported in the literature involving different parts of the body including lymph nodes, central nervous system [4], pleura, lung mass, pleural mass [5, 6].

Development of paradoxical pleural effusion as a manifestation of TB IRIS has been reported to occur in 1.5% to 23% of TB cases [2, 3]. The severity of reported cases varies from asymptomatic to severe [7]. Paradoxical pleural effusion as a manifestation of TB IRIS can occur during treatment for pleural TB with worsening of effusion at the same site or on the contralateral side [8, 9]; it has also been reported during TB treatment at other sites like cervical lymph node [10]. The possible differential diagnosis for worsening pleural effusion in such cases includes drug‐resistant TB, INH‐induced lupus, and malignant pleural effusion [11].

Though there is a lack of guidelines in the management of TB IRIS, steroids have been used in patients with TB IRIS, especially in HIV‐infected individuals [12]. Done et al. reported two cases of TB IRIS in 2 HIV‐negative bacteriologically confirmed pulmonary TB cases who were successfully treated with prednisolone given for a total duration of 37 days and 3 months [13]. Jung et al. reported TB IRIS to be present in 23% (32 of 139) cases of Pleural TB, 18 of whom needed treatment; the treatments included drainage only (in 7), thoracotomy (in 4), and seven patients needed steroids. Among the patients who were given prednisolone 0.5 mg/kg, five responded within 2 weeks (in 3).

Despite Ethiopia being among the top 30 TB affected areas in the world, to the authors' knowledge, there seems to be no published study on TB IRIS in the pleura in non‐HIV infected individuals. There is also no large published prevalence study or meta‐analysis from sub‐Saharan Africa [14].

Note that in this case the level of IL was not measured as it is not available in the country. ADA was also not available; serial measures of inflammatory markers were also not done. Lights criteria were not included because serum protein was missing from the chart. The case shows limitations of diagnostics in an area where TB is common. Other limitations include lack of biopsy due to consent. However, the clinical improvement and stability go with the diagnosis of IRIS.

## 3 Conclusion

In this case report a massive pleural effusion developed after commencement of TB treatment contrary to the expected improvement. Clinicians may consider another differential diagnosis such as malignancy or drug resistance which requires extensive tests with potentially unnecessary treatment. Having IRIS as a diffrential diagnosis in such cases could prevent the mentioned consequences as this can potentially be treated with a short course of steroid as in our patient. As TB still remains a global public health challenge with IRIS occurring in non‐HIV positive patients further studies are required to understand this phenomenon and develop evidence‐based guideline; which is currently lacking.

## Author Contributions

**Selam Bogale Gissa:** conceptualization, data curation, formal analysis, investigation, writing – original draft, writing – review and editing. **Alemayehu Girma Gemechu:** conceptualization, data curation, writing – original draft, writing – review and editing. **Selamawit Tilahun:** conceptualization, data curation, writing – review and editing.

## Funding

The authors have nothing to report.

## Ethics Statement

Yekatit 12 Hospital Medical College Research and Publication Office gave ethical approval (Protocol number 251/23).

## Consent

For this case report, written informed consent was obtained from the patient for publication of this case report and any accompanying images. A copy of the written consent is available for review by the Editor‐in‐Chief of this journal.

## Conflicts of Interest

The authors declare no conflicts of interest.

## Data Availability

Data supporting this case report will be available with the corresponding author upon request.

## References

[1] L. C. K. Bell , R. Breen , R. F. Miller , M. Noursadeghi , and M. Lipman , “Paradoxical Reactions and Immune Reconstitution Inflammatory Syndrome in Tuberculosis,” International Journal of Infectious Diseases 32 (2015): 39–45, 10.1016/j.ijid.2014.12.030. 25809754

[2] S. Hammi , N. Zemed , K. Bouti , and J. E. Bourkadi , “Paradoxical Reactions During Antituberculosis Therapy: A Single‐Center Prospective Analysis,” International Journal of Medical Sciences 2, no. 2 (2015), 10.15342/ijms.v2i2.75.

[3] J. W. Jung , J. W. Shin , J. Y. Kim , et al., “Risk Factors for Development of Paradoxical Response During Anti‐Tuberculosis Treatment in HIV‐Negative Patients With Pleural Tuberculosis,” Tohoku Journal of Experimental Medicine 223, no. 3 (2011): 199–204, 10.1620/tjem.223.199. 21372521

[4] L. Balboa , “Cerebral Tuberculoma as Paradoxical Reaction,” Journal of Neurosciences in Rural Practice 3, no. 3 (2012): 350–351. 23188994 10.4103/0976-3147.102622 PMC3505333

[5] Z. Dong , W. Zhang , W. Sun , et al., “Paradoxical Development of Pleural Based Masses in Patients With Pleural Tuberculosis During Treatment: A Clinical Observational Study in China,” BMC Pulmonary Medicine 22 (2022): 126, 10.1186/s12890-022-01910-6. 35379218 PMC8981736

[6] D. Varona Porres , E. Pallisa , A. L. Sánchez , and Ó. Persiva , “Solitary Pleural Nodule: A Late Paradoxical Reaction to Antituberculosis Treatment,” Radiología 62, no. 5 (2020): 411–414. 32381376 10.1016/j.rx.2020.01.009

[7] K. Jeon , W. I. Choi , J. S. An , et al., “Paradoxical Response in HIV‐Negative Patients With Pleural Tuberculosis: A Retrospective Multicentre Study,” International Journal of Tuberculosis and Lung Disease 16, no. 6 (2012): 846–851, 10.5588/ijtld.11.0642. 22507441

[8] A. H. Ahmed and S. Goddard , “Contralateral Paradoxical Response to Chemotherapy in Tuberculous Pleural Effusion,” Sudan Journal of Medical Sciences 3, no. 1 (2008): 63–65.

[9] P. Ish , S. Chakrabarti , and D. Bhattacharya , “Flummoxing Paradox of Contralateral Pleural Effusion Developing During Successful Drug Treatment of a Tubercular Pleural Effusion,” Astrocyte 3, no. 2 (2016): 104–106, 10.4103/2349-0977.197216.

[10] A. Bhattacharya and S. Mukherjee , “Paradoxical Reaction in the Form of Pleural Effusion After Onset of Anti‐Tuberculous Medication for Tubercular Lymphadenitis,” Clinical Medicine 17, no. 2 (2017): 143–145. 28365625 10.7861/clinmedicine.17-2-143 PMC6297614

[11] V. Varenika and P. Blanc , “A Patient on RIPE Therapy Presenting With Recurrent Isoniazid‐Associated Pleural Effusions: A Case Report,” Journal of Medical Case Reports 5 (2011): 558, 10.1186/1752-1947-5-558. 22129471 PMC3296633

[12] S. J. E. Beishuizen and S. E. Geerlings , “Paradoxical Reactions in Tuberculosis Treatment,” Netherlands Journal of Medicine 67, no. 10 (2009): 327–331. 19915226

[13] M. M. Done , O. W. Akkerman , W. Al Kailany , et al., “Corticosteroid Therapy for the Management of Paradoxical Inflammatory Reaction in Patients With Pulmonary Tuberculosis,” Infection 48 (2020): 641–645, 10.1007/s15010-020-01430-7. 32333368 PMC7394936

[14] World Health Organization , Global Tuberculosis Report 2024 (World Health Organization, 2024), https://www.who.int/teams/global‐tuberculosis‐programme/tb‐reports/global‐tuberculosis‐report‐2024.

